# Enhanced Activity of Alkali-Treated ZSM-5 Zeolite-Supported Pt-Co Catalyst for Selective Hydrogenation of Cinnamaldehyde

**DOI:** 10.3390/molecules28041730

**Published:** 2023-02-11

**Authors:** Shibo Cheng, Shan Lu, Xiang Liu, Gao Li, Fei Wang

**Affiliations:** 1 Pittsburgh Institute (SCUPI), Sichuan University, Chengdu 610065, China; 2 Advanced Catalysis and Green Manufacturing Collaborative Innovation Center, School of Petrochemical and Engineering, Changzhou University, Changzhou 213164, China; 3 Hunan Drug Inspection Center, Hunan Institute for Drug Control, Changsha 410013, China; 4 Dalian Institute of Chemical Physics, Chinese Academy of Sciences, Dalian 116023, China

**Keywords:** Pt-Co, ZSM-5, selective hydrogenation, cinnamaldehyde, cinnamyl alcohol

## Abstract

A bimetallic Pt8Co1 supported on alkali-treated ZSM-5 zeolite (ZSM-5-AT) was prepared through the impregnation method. The structure and surface properties of the catalysts were characterized by X-ray diffraction (XRD), transmission electron microscopy (TEM), N_2_-sorption and X-ray photoelectron spectroscopy (XPS) as well as temperature-programmed desorption of NH_3_ (NH_3_-TPD) and temperature-programmed reduction of H_2_ (H_2_-TPR). The TEM images present that the bimetallic Pt8Co1 nanoparticles with a mean particle size of 4–6 nm were uniformly dispersed on the alkali-treated ZSM-5 zeolite. The bimetallic Pt8Co1/ZSM-5-AT catalyst exhibited an extraordinary COL selectivity of 65% at a >99% CAL conversion efficiency, which showed a much higher catalytic performance (including the activity and selectivity) than the monometallic Pt/ZSM-5-AT and Co/ZSM-5-AT catalysts in the selective hydrogenation of cinnamaldehyde (CAL) to cinnamyl alcohol (COL) using hydrogen as reducing agent. The high catalytic activity of the bimetallic catalyst was attributed to the higher electron density of Pt species and more acidic sites of the alkali-treated ZSM-5 zeolite support. The recovery test showed no obvious loss of its initial activity of the Pt8Co1/ZSM-5-AT catalyst for five times.

## 1. Introduction

**Scheme 1:**
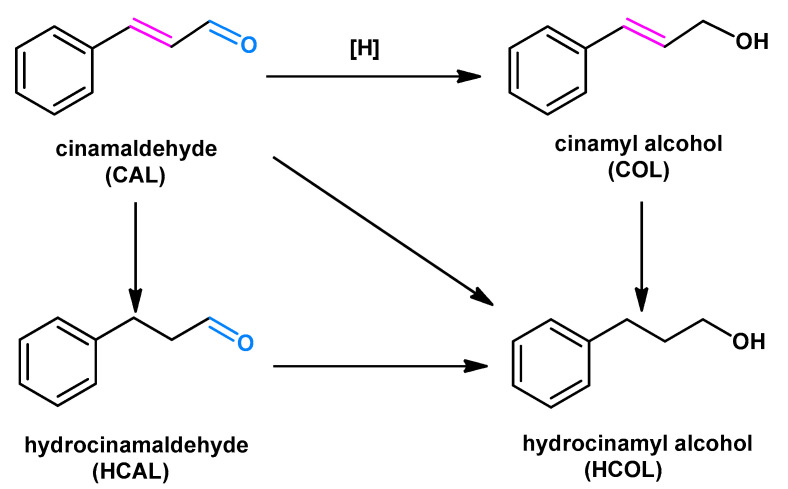
The cinnamaldehyde transformation to cinnamyl alcohol (COL), hydrocinamaldehyde (HCAL), and hydrocinamyl alcohol (HCOL) upon the selective hydrogenations.

The selective hydrogenation of α,β-unsaturated aldehydes to corresponding α,β-unsaturated alcohols has attracted remarkable attention, due to the importance of its product in fine-chemicals, pharmaceuticals and fragrances [1,2,3]. Among them, cinnamaldehyde (CAL) has been usually adopted as an important and representative reactant for the selective hydrogenation and investigated in recent decades [4,5]. However, the selective hydrogenation of CAL can lead to different products, and thus, the controlled selectivity was also challenging because of multifunctional groups [6,7], as depicted in Scheme 1. Moreover, owing to the conjugated C=O and C=C bonds, the selective hydrogenation of C=O bonds to cinnamyl alcohol (COL) was not thermodynamically favorable. Therefore, the highly selective hydrogenation of CAL to COL was still a conundrum [8,9,10].

In order to solve this problem, effective catalysts were highly desirable to raise the selectivity of α,β-unsaturated alcohols [11]. According to previous literature, metallic Pt-based catalysts were the most efficient catalyst for the selective hydrogenation of CAL to COL [12,13,14]. Regulation of the particle size and metal dispersion degree can improve the catalytic activity, but Pt nanoparticles still showed unsatisfied COL selectivity [15,16,17]. Due to geometric and electronic effects, satisfactory catalytic property can be gained by introducing a second metal and form alloy structure [18], e.g., Pt-Fe [19,20], Pt-Ni [21], and so on.

Alternatively, an appropriate support (such as oxides, carbons, and zeolites) can adjust the electronic properties of Pt species via their interaction, and consequently, it promotes the reaction activity, selectivity, and recycle stability [22]. In our previous work, the optimal electronic Pt-Ce-La composite support interactions on the redox sites, the excellent catalytic performance was achieved [23]. The same example, such as Wang et al. reported a Pt/mesoTiO_2_-SiO_2_-M catalyst, showed excellent COL selectivity at almost complete CAL conversion, because electrons transfer from the mesoTiO_2_-SiO_2_-M to Pt, leading to a high density of electrons on the Pt surfaces. Hence, the C=O bond of CAL can be preferentially adsorbed and activated [24].

Due to good hydrothermal stability, a wide range of Si/Al ratio, three-dimensional channels, good shape selectivity, large specific surface area and pore volume, ZSM-5 zeolites are widely used as support of catalysts in petrochemical, fine chemical, and other industries [25]. Bifunctional zeolite-supported metal catalysts, typically containing both metal sites and zeolite acid sites, perform (de)hydrogenation of hydrocarbons and isomerization/cracking of the dehydrogenated hydrocarbon species, respectively, which are highly important for fine chemical synthesis such as chemoselective hydrogenation of α,β-unsaturated aldehydes to corresponding α,β-unsaturated alcohols [26]. To achieve high activity, selectivity, and stability of the catalysts, many works focus on promoting the dispersion of the metal nanoparticles or adjusting the pore structure of ZSM-5 zeolites. For instance, Li et al. [19] prepared a HPZSM-5 with hierarchical porous structure by partial desilication of commercial ZSM-5, which was proven to be an excellent support for Pt-Fe in the selective hydrogenation of CAL to yield COL. On the one hand, partial desilication of ZSM-5 strengthen the interaction of Pt-support compared with conventional ZSM-5. On the other hand, doping of Fe obviously enhanced the catalyst activity via electron transfer from Fe species to Pt atoms. In addition, according to previous work, mild alkali treatment of ZSM-5 zeolites can reduce the strong acid sites and the pore structure was not changed obviously, but severe alkali treatment can create new mesopores and the stronger acid sites [27].

Herein, the monometallic (Pt and Co) and bimetallic (Pt-Co) supported on alkali-treated ZSM-5 catalysts (Pt/ZSM-5-AT, Co/ZSM-5-AT and PtCo/ZSM-5-AT) were successfully synthesized via an impregnation method for the selective hydrogenation of CAL to produce COL. The structure and physicochemical properties of these catalysts were characterized by X-ray diffraction (XRD), transmission electron microscopy (TEM), N_2_-sorption and X-ray photoelectron spectroscopy (XPS) as well as temperature-programmed desorption of NH_3_ (NH_3_-TPD) and temperature-programmed reduction of H_2_ (H_2_-TPR). The catalytic performances of the Pt/ZSM-5-AT, Co/ZSM-5-AT and PtCo/ZSM-5-AT catalysts were investigated. The bimetallic Pt8Co1/ZSM-5-AT catalyst exhibited a much higher catalytic performance (including the activity and selectivity) than the monometallic Pt/ZSM-5-AT and Co/ZSM-5-AT catalysts in the selective hydrogenation of CAL to COL using hydrogen as reducing agent. Moreover, a correlation was discussed between the structure of catalysts and catalytic performance. The recycle stability of the catalyst was also evaluated. Finally, the tentative reaction mechanism was given.

## 2. Results and Discussion

### 2.1. Textural and Structural Properties of the Alkali-Treated ZSM-5-Supported Catalysts

**Figure 1:**
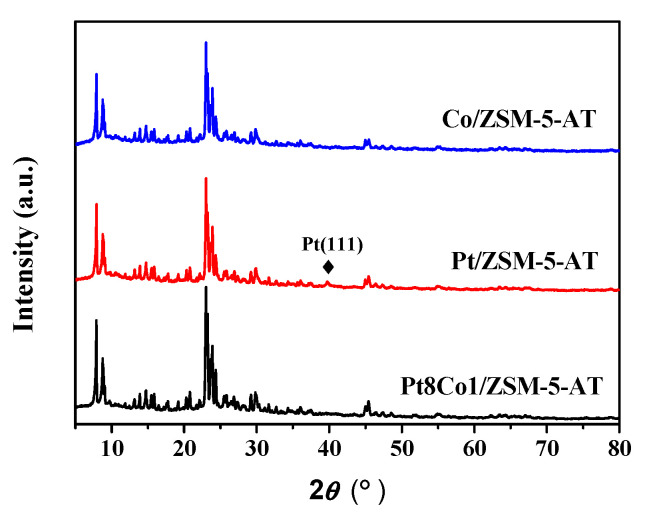
XRD patterns of the as-prepared Pt/ZSM-5-AT, Co/ZSM-5-AT and Pt8Co1/ZSM-5-AT catalysts.

Figure 1 showed the XRD patterns of the as-prepared Pt/ZSM-5-AT, Co/ZSM-5-AT, and Pt8Co1/ZSM-5-AT catalysts. All samples showed a serial of peaks at ranges of 2θ 7° to 9° and 23° to 25°, which were attributed to the characteristic peaks of ZSM-5 zeolite [27], which implied that the crystalline structure of ZSM-5 did not obviously change after the Pt and/or Co supported on ZSM-5. A tiny diffraction peak at 39.5° was found at Pt/ZSM-AT sample, which corresponded to the Pt(111) of the cubic Pt. Additionally, it is feasible that Co might be present in the amorphous form and thus is not detected. Additionally, note that there was no characteristic peak of Pt phase detected in the XRD patterns of the as-prepared Pt8Co1/ZSM-5-AT catalysts, which may be due to relatively low loading amounts or high dispersion on the ZSM-5-AT surfaces.

**Figure 2:**
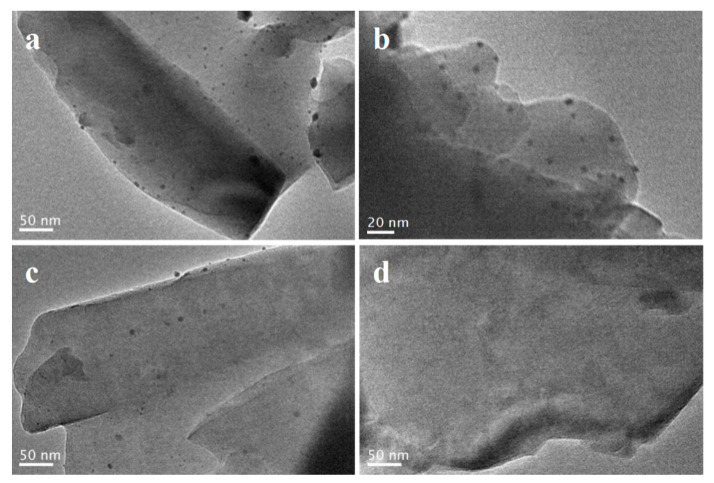
TEM images of the as-prepared Pt8Co1/ZSM-5-AT (**a**,**b**), Pt/ZSM-5-AT (**c**) and Co/ZSM-5-AT (**d**) catalysts.

Next, the location of metal particles in the spent catalysts was also examined by TEM method. Figure 2 displayed the TEM images of these ZSM-5-AT-supported metal catalysts. As seen from Figure 2a,b, the PtCo nanoparticles on the ZSM-5-AT support display an average size of 4–5 nm measured from randomly counting 100 particles and the bimetallic PtCo nanoparticles were highly dispersed on the surface of ZSM-5-AT support. Compared with the as-prepared Pt8Co1/ZSM-5-AT catalysts (Figure 2c), the relative bigger Pt particles with size of 6–7 nm were found for Pt/ZSM-5-AT catalyst, demonstrating that the existence of Co was favorable for the distribution of metallic Pt particles. The small size and high uniformity of the PtCo bimetallic nanoparticles in Pt8Co1/ZSM-5-AT would be beneficial to offer more active reaction sites and enhance the catalytic activity. In addition, as shown in Figure 2d, no Co nanoparticles were observed in Co/ZSM-5-AT catalysts. These results were also consistent with the XRD pattern.

**Figure 3:**
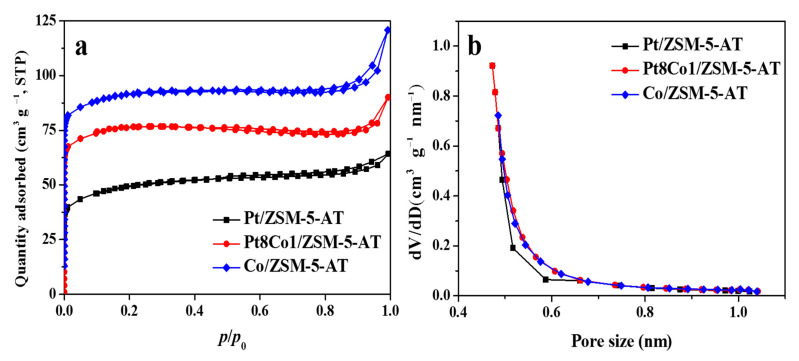
The N_2_ adsorption–desorption curve (**a**) and pore diameter distribution (**b**) of the as-prepared Pt8Co1/ZSM-5-AT, Pt/ZSM-5-AT and Co/ZSM-5-AT catalysts.

Metal loaded and composition results of as-prepared Pt8Co1/ZSM-5-AT, Pt/ZSM-5-AT and Co/ZSM-5-AT catalysts are summarized in Table 1. N_2_ adsorption–desorption isotherms and pore size distribution curves were presented in Figure 3a. It can be seen that all catalysts present typical isotherms of type-I. It implied the presence of their pore sizes was almost distributed in the arrange of 0.5 nm to 2.0 nm [28]. The physical parameters of these catalysts are summarized in Table 1. BET analysis was performed to measure the surface areas of the as-prepared Pt8Co1/ZSM-5-AT, Pt/ZSM-5-AT, and Co/ZSM-5-AT catalysts. The BET surface area of Co/ZSM-5-AT and Pt/ZSM-5-AT was 242.1 m^2^ g^−1^ and 247.4 m^2^ g^−1^, respectively, whereas that of Pt8Co1/ZSM-5-AT was 227.8 m^2^ g^−1^. The decrease in surface area of Pt8Co1/ZSM-5-AT could be due to the incorporation of Co and Pt on the ZSM-5-AT surface. The pore size distribution of the Pt8Co1/ZSM-5-AT, Pt/ZSM-5-AT, and Co/ZSM-5-AT catalysts, determined by the Barrett–Joyner–Halenda (BJH) method, were almost same (Figure 3b), indicating that the Pt8Co1, Pt, and Co particles should only be anchored on the surface of the alkali-treated ZSM-5 support.

**Figure 4:**
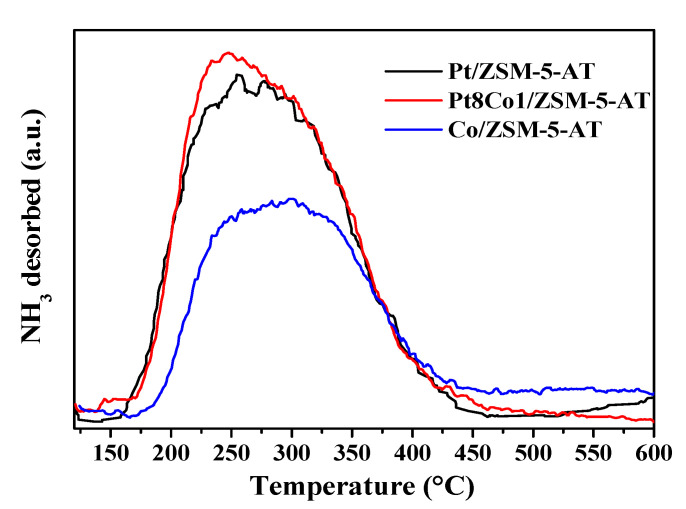
NH_3_-TPD profiles of the as-prepared Pt8Co1/ZSM-5-AT, Pt/ZSM-5-AT and Co/ZSM-5-AT catalysts.

Further, to investigate the acidity of the as-prepared Pt8Co1/ZSM-5-AT, Pt/ZSM-5-AT, and Co/ZSM-5-AT catalysts, the NH_3_-TPD analyses were carried out. Based on previous reports, ZSM-5 support exhibited two NH_3_ desorption peaks at 241 °C and 445 °C, which can be assigned to a weak acid site and a strong acid site on the surface of ZSM-5 zeolite, respectively [29]. As displayed in Figure 4, after loading Pt and/or Co, all catalysts only showed one ammonia desorption broad peak centered at about 250 °C (180 °C to 400 °C), which was attributed to the weak acid sites. It was worth noting that no high-temperature desorption peak was observed, showing that the strong acid center on zeolite should be preferentially neutralized by the introduction of metals. Comparatively, the amount of acid increased obviously with the increase in the addition of Co species. As shown in Table 1, the weak acid content of Pt/ZSM-5-AT was 263.9 µmol·g^−1^ (Table 1, entry 1), while the amount of Pt8Co1/ZSM-5-AT reached 284.3 µmol·g^−1^ (Table 1, entry 2). These findings demonstrated that the addition of Co could change the acid character of the catalyst effectively. According to the previous literature [30], enhanced support acidity of catalysts would lead to an enhanced activity in hydrogenation.

### 2.2. Surface Properties

**Figure 5:**
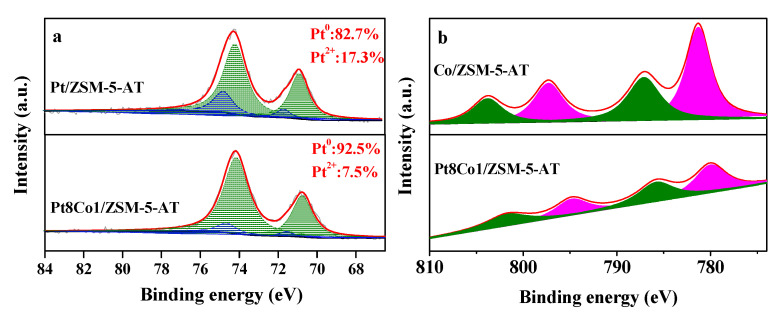
(**a**) Pt 4f XPS spectra of as-prepared Pt8Co1/ZSM-5-AT and Pt/ZSM-5-AT catalysts. (**b**) Co 2p XPS spectra of as-prepared Pt8Co1/ZSM-5-AT and Co/ZSM-5-AT catalysts.

The XPS was a surface-sensitive technology to confirm the Pt locations and their oxidation states. As depicted in Figure 5a, the Pt 4f spectra over Pt8Co1/ZSM-5-AT and Pt/ZSM-5-AT catalysts including four deconvoluted peaks at 71.6 eV, 74.9 eV, 72.5 eV, and 75.9 eV could be assigned to the Pt^0^ and Pt^2+^ species, respectively [31]. Further, we calculated and determined the amount of the surface Pt species (Pt^0^ and Pt^2+^) based on the area of the peaks. As also shown in the Figure 5 inset, the relative amount of Pt^0^ species and Pt^2+^ species in Pt/ZSM-5-AT catalyst was 82.7% and 17.3%, respectively. Additionally, for Pt8Co1/ZSM-5-AT catalyst, the relative amount of metallic Pt^0^ and Pt^2+^ species was 92.5% and 7.5%. The amount of Pt^0^ species in Pt8Co1/ZSM-5-AT was higher by about 10% than that of Pt/ZSM-5-AT catalyst. It indicated that there was strong interaction between Pt and Co species and electron transfer from Co species to Pt species occurred [32]. Thus, we here speculated that highly active electron-rich Pt^0^ sites in Pt8Co1/ZSM-5-AT catalyst stabilized by strong metal–support interaction (SMSI) would effectively adsorb and activate molecular H_2_ and C=O bond in CAL. In addition, the Co 2p peaks of the prepared samples are plotted in Figure 5b. The XPS spectra of as-prepared Pt8Co1/ZSM-5-AT and Co/ZSM-5-AT catalysts showed two peaks at 778.9 eV (2p3/2) and 794.eV (2p1/2), characteristic of metallic Co (Co^0^). A shift toward low binding energy for Pt8Co1/ZSM-5-AT was observed in Co 2p spectra compared to Co/ZSM-5-AT, most likely resulting from the electron transfer between Co and Pt [33].

**Figure 6:**
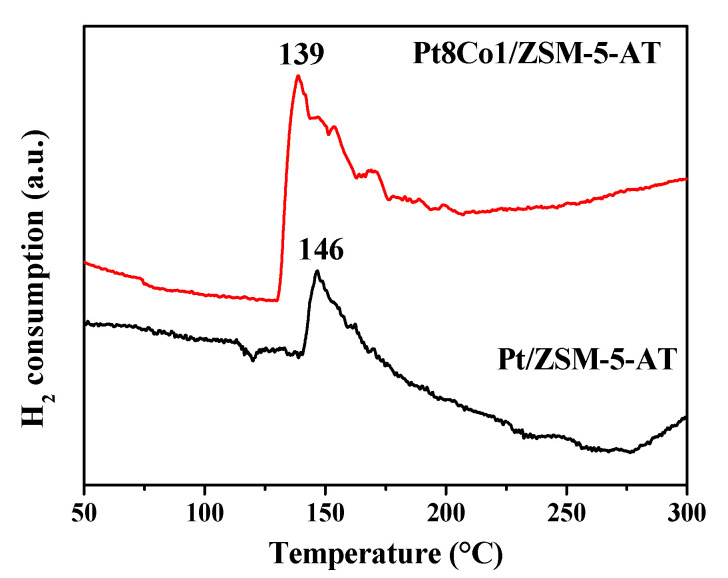
The H_2_-TPR profiles of the as-prepared Pt8Co1/ZSM-5-AT and Pt/ZSM-5-AT catalysts.

The strong interaction between Pt and Co could significantly affect the redox properties. Further, the TPR measurements were carried out to investigate the effect of Co addition on the catalyst reducibility. As shown in Figure 6, the Pt/ZSM-5-AT produced two peaks: one peak due to hydrogen spillover (120 °C) and the other one due to the reduction of Pt^2+^ species to metallic Pt^0^ species (at ~146 °C) [34]. It should be noted that the hydrogen spillover peak was produced only by the Pt/ZSM-5-AT catalyst, indicating that the as-formed Pt-Co bimetallic structure inhibited the hydrogen spillover, and the Co atoms were even dispersed into the bimetallic Pt rich particles. More importantly, the bimetallic Pt8Co1/ZSM-5-AT catalyst showed a sharp peak (around 139 °C), which was at a lower temperature and much more intense than monometallic Pt/ZSM-5-AT catalyst (~146 °C). It was rational to assume that hydrogen that dissociated on the Co surface could migrate to the surface of Pt and support, resulting in Pt being reduced at much lower temperatures. Furthermore, the total H_2_ uptake of Pt8Co1/ZSM-5-AT catalyst was obviously higher than the sum of the H_2_ uptakes of Pt/ZSM-5-AT catalyst. These results indicated that PtCo bimetallic systems showed more hydrogen consumption, which may correspond to the intense metal–metal and metal–support interactions [23,35].

### 2.3. Chemoselective Hydrogenation of COL

**Figure 7:**
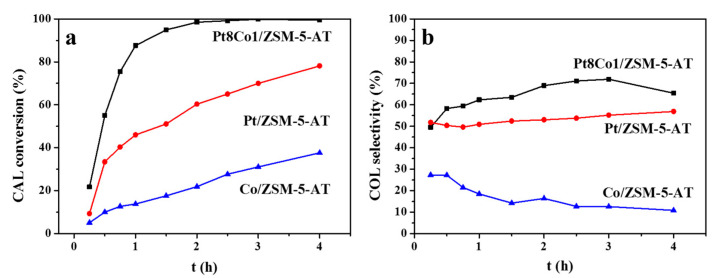
The catalytic results for the selective hydrogenation of CAL over the Pt8Co1/ZSM-5-AT, Pt/ZSM-5-AT, and Co/ZSM-5-AT catalysts: (**a**) CAL conversion and (**b**) COL selectivity. Reaction conditions: 200 mg of catalyst, 4 mmol of CAL, P_H2_ = 2 MPa, 20 mL of ethanol, at 100 °C, with a stirring rate of 1000 rpm.

The selective hydrogenation of CAL to COL was performed on alkali-treated ZSM-5 zeolite-supported Pt, Co, and PtCo particle catalysts. From Figure 7, it can be seen that the addition of Co to Pt/ZSM-5-AT catalyst obviously improved catalytic activity and the selectivity for COL. Table 2 listed the detailed catalytic results obtained with the Pt8Co1/ZSM-5-AT, Pt/ZSM-5-AT, and Co/ZSM-5-AT catalysts. Pt/ZSM-5-AT catalyst afforded an unsatisfactory CAL conversion of 60.3% and a COL selectivity of 53% (Table 2, entry 2). In contrast, the Pt8Co1/ZSM-5-AT catalyst exhibited much higher catalytic activity than other two homo-metal catalysts. Both the CAL conversion and COL selectivity were obviously increased. As a result, a 93.7% CAL conversion with a 68.9% COL selectivity was achieved with the Pt8Co1/ZSM-5-AT catalyst (Table 2, entry 1). Additionally, homo Co supported on ZSM-5-AT catalyst was not efficient for this reaction (21.9% CAL conversion and 16.4% COL selectivity). Previous characterization results showed that Pt valency almost remained intact after the addition of Co. Therefore, the enhanced CAL conversion and COL selectivity might be assigned as the change in the electronic environment on the metal surface, leading to modifications of the electronic structure by the metal–metal interaction. Thus, the superior catalytic activity of Pt8Co1/ZSM-5-AT in the hydrogenation reactions is mainly attributed to be the high population of the metallic Pt^0^ species and more acidic sites in catalyst.

**Figure 8:**
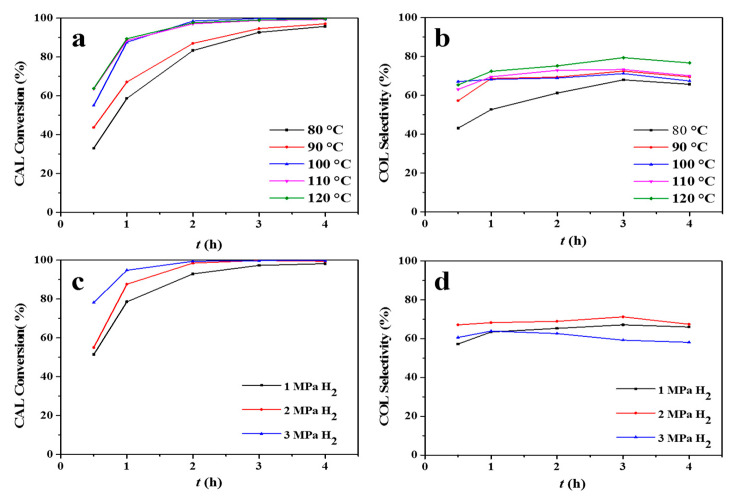
Effect of (**a**,**b**) reaction temperature (2 MPa H_2_, 0–4 h) and (**c**,**d**) H_2_ pressure (100 °C, 0–4 h) on CAL conversion efficiency and COL selectivity over the Pt8Co1/ZSM-5-AT catalyst. Reaction conditions: 200 mg of catalyst, 4 mmol of CAL, 20 mL of ethanol, with a stirring rate of 1000 rpm.

Next, the effect of reaction temperature and H_2_ pressure on CAL conversion efficiency and COL selectivity using the Pt8Co1/ZSM-5-AT catalyst was also investigated (Figure 8). As shown in Figure 8a,b, an appreciable amount (58%) of CAL was converted at 80 °C with a selectivity of 52% in one hour time at a H_2_ pressure of 2 MPa. On increasing the temperature from 80 °C to 100 °C, an 88.7% CAL conversion with a 67.2% selectivity towards COL was achieved. Increasing the reaction temperature to 120 °C does not obviously affect the CAL conversion and COL selectivity. In addition, from Figure 8c,d, increasing the hydrogen pressure from 1 MPa to 3 MPa resulted in an increase in the conversion of CAL from 77% to 94%, indicating that the activity was directly related to the H_2_ pressure, effectively presenting a first-order dependency. Moreover, the COL selectivity remained at 60% in the range of pressures.

**Figure 9:**
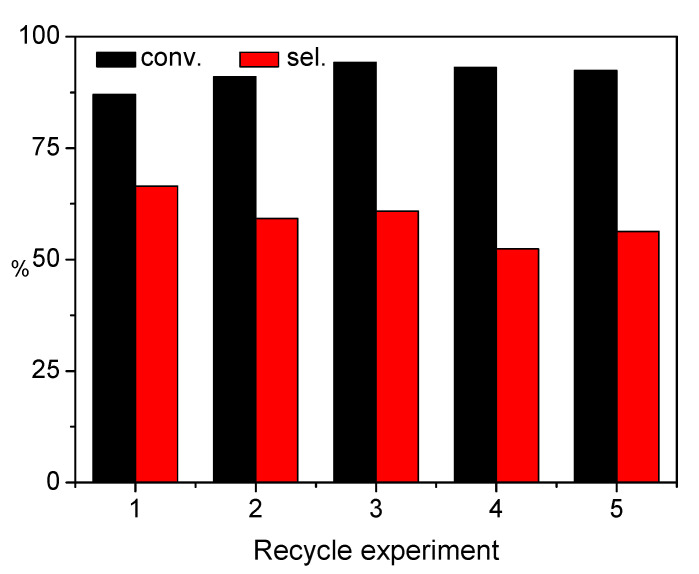
Reusability of the Pt8Co1/ZSM-5-AT catalyst toward the selective hydrogenation of CAL. Reaction conditions: 200 mg of catalyst, 4 mmol of CAL, P_H2_ = 2 MPa, 20 mL of ethanol, at 100 °C, for 2 h, with a stirring rate of 1000 rpm.

Moreover, the recycle stability of the Pt8Co1/ZSM-5-AT catalyst was also investigated. As clearly seen in Figure 9, the Pt8Co1/ZSM-5-AT catalyst can be recycled for five times without obvious loss in CAL conversion or COL selectivity. To test the catalyst stability, the filtrate was detected using the ICP-AES method. As a result, the leached Pt amount in the filtrate is below the detection limit of ICP-AES, suggesting that the Pt8Co1 is very robust during the hydrogenation processes.

### 2.4. Tentative Reaction Mechanism

Based on the catalytic studies, a possible reaction mechanism over the alkali-treated ZSM-5 zeolite-supported Pt8Co1 catalysts was briefly presented here. Firstly, the CAL reactant should be chemo-absorbed on the surface of Pt8Co1 particles. The addition of Co improves the electron-rich Pt^0^ species originating from strong metal–support interaction with alkali-treated ZSM-5 zeolite. Then, the hydrogen can dissociate into H^−^ and H^+^ over the surface of metallic Pt^0^ species. Secondly, the H^−^ nucleophile will attack the C=O group of CAL to generate the COL product, which will be desorbed from PtCo particles upon the completion of hydrogenation. The high population of the metallic Pt^0^ species promotes the hydrogenation reactions, and the alkali-treated ZSM-5 zeolite would accelerate COL production and display a high formation of COL in the prolonged hydrogen duration to obtain a high COL selectivity.

## 3. Materials and Methods

### 3.1. Materials

All chemicals, including the solvents, were commercially available as reagent grade and used as received without further purification. Co(NO_3_)_2_·6H_2_O (98%), H_2_PtCl_6_·6H_2_O (98%), NaOH (99%), CAL (98%), and ethanol (99%) were purchased from Adamas-beta^®^ (Shanghai, China). HZSM-5 with a SiO_2_/Al_2_O_3_ molar ratio of 600 was purchased from Nankai University Catalyst Ltd. (Tianjin, China). All the gases were purchased from Dalian gas company (Dalian, China). Ultrapure water was purified with a Barnstead Nanopure Di-water TM system (Dubuque, IA, USA). All glassware was thoroughly cleaned with aqua regia (37 wt% HCl: HNO_3_ = 3:1 by volume), rinsed with copious nano-pure water, and then dried in an oven before use.

### 3.2. Catalyst Preparation

An amount of 0.5 g of as-received ZSM-5 zeolite was dispersed in 5 mL of 0.1 M NaOH solution under stirring for 4 h. The solids were collected by filtration and washed with water for three times to remove the excess NaOH and salts. To prepare the Pt8Co1/ZSM-5-AT catalyst, the desired amounts of Co(NO_3_)_2_·6H_2_O and H_2_PtCl_6_·6H_2_O were added into the alkali-treated ZSM-5 suspension. The resultant solution was heated at 60 °C with continuous stirring. All the catalysts were dried at 80 °C for 8 h and calcined at 400 °C for 4 h under static air in a muffle furnace to obtain the 1% Pt8Co1/ZSM-5-AT catalyst. Additionally, the monometallic-supported ZSM-5 catalysts of 1% Pt/ZSM-5-AT and 1% Co/ZSM-5-AT were achieved by the same method.

### 3.3. Catalyst Characterization

XRD (X-ray diffraction) patterns were recorded on a Rigaku D/MAX2500/PC (Rigaku Co., Tokyo, Japan) diffractometer using Cu Ka radiation (40 kV, 200 mA), at a scanning rate of 10° min^−1^ in the range of 2θ from 5° to 60°. The transmission electron microscopy (TEM) images were recorded on a FEI Tecnai G2 Spirit microscope (Tokyo, Japan) operated at 120 kV. The specimens were prepared by ultrasonically dispersing the sample into ethanol, depositing droplets of the suspensions onto a carbon-enhanced copper grid, and drying them in air. N_2_ adsorption–desorption isotherms were measured at −196 °C using a Micromeritics ASAP 2020 analyzer (Norcross, GA, USA). Prior to the analysis, the samples were degassed (1.33 Pa) at 150 °C for at least 4 h. The specific surface area was calculated according to the Brunauer–Emmet–Teller (BET) method, and pore size distribution was determined by the Barret–Joyner–Halenda method. Temperature-programmed desorption of NH_3_ (NH_3_-TPD) on the samples was performed on a micromeritics Autochem II chemosorption analyzer (Norcross, GA, USA). Then, 100 mg of the sample was loaded into a quartz reactor and heated in a flow of He gas (30 mL min^−1^) at 400 °C for 1 h to remove the adsorbed pollutants and then exposed under a flow of H_2_ (30 mL min^−1^) for 1 h. After the reactor was cooled to room temperature under a flow of He gas, the sample was exposed to a mixture of 15 vol.% NH_3_/He (30 mL min^−1^) for 1 h at 10 °C. After being purged with He (30 mL min^−1^) for 2 h, the sample was heated to 900 °C at a rate of 5 °C min^−1^. The concentration of desorbed ammonia was analyzed by a TCD detector connected to the TPD system. X-ray photoelectron spectroscopy (XPS) measurements were performed within a UHV system (base pressure: 5 × 10^−10^ mbar), equipped with a Phoibos 100 hemispherical analyzer (Saint Louis, Missouri, USA) and XR 50 X-ray source (SPECS GmbH). The catalyst powder samples were placed on transferrable sample holders using UHV-compatible conductive carbon tape. Spectra were measured at room temperature using Al-Kα radiation (1486.61 eV) and an electron emission angle of 0°, with the analyzer operated in “large area” transmission mode. Using CasaXPS, all spectra were referenced to the C 1 s signal (C-C, 284.6 eV). Subsequently, peaks were fitted after Shirley background subtraction utilizing Gauss–Lorentz sum functions, with peak positions and full width half maxima (FWHM) left unconstrained. For Pt 4f, and a peak area ratio of 4f_5/2_:4f_7/2_ = 3:4 was assumed (according to NIST XPS database). Temperature-programmed reduction of H_2_ (H_2_-TPR) experiments were tested on Micromeritics AutoChem1 II 2920 (Norcross, GA, USA) with a TCD (thermal conductivity detector). Typically, 50 mg of the samples were loaded into a linear quartz tube along with quartz wool. Before analysis, the samples were pre-treated with a flow of He gas (30 mL min^−1^) at 400 °C for 1 h to clean the sample surface, and then the sample was exposed at the same temperature for 1 h under a flow of H_2_ gas (30 mL min^−1^). After cooled to −50 °C in a flow of He (30 mL min^−1^), reduction was carried out under a flow of 5% H_2_/He (30 mL min^−1^) at a heating rate of 10 °C min^−1^ when the signal is stale. The amount of H_2_ consumed was determined from the TCD signal intensities calibrated. The amounts of Pt and Co were determined using Inductively Coupled Plasma-Mass Spectrometer (ICP-MS).

### 3.4. Catalyst Evaluation

The selective hydrogenation of CAL was conducted in a stainless-steel autoclave (50 mL). Typically, the reaction mixture contained 0.5 mL of CAL, 20 mL of ethanol (solvent) and 200 mg of catalyst. During the hydrogenation, catalysts were suspended in solution by violent stirring. The reaction was performed at 80 °C to 110 °C for a certain period (0–8 h) under 3.0 MPa H_2_ pressure. At the end of the reaction, the reactor was cooled to the ambient temperature, and the catalysts were separated from the reaction mixture. The product of each hydrogenation reaction was analyzed by Haixin GC-950 gas chromatography (HP-5 capillary column, Hebei, China). For the recycle tests, the Pt8Co1/ZSM-5-AT catalysts were collected by filtration and washed with water and ethanol. Additionally, it was then added to the fresh reaction mixtures under the same reaction conditions. The conversion of CAL (X_CAL_) and the selectivity to corresponding COL (S_COL_) were calculated as:

X_CAL_ = (C_0_ − C_1_)/C_0_ × 100% (1)

S_COL_ = C_COL_/(C_0_ − C_1_) × 100% (2)

where C_0_, C_1_, and C_COL_ represent the initial concentration of CAL, and concentrations of CAL and COL after the catalytic hydrogenations, respectively.

## 4. Conclusions

In summary, bifunctional Pt-Co bimetallic supported on alkali-treated ZSM-5 zeolite catalysts were prepared. Further, the catalysts were applied for the selective hydrogenation of cinnamaldehyde to cinnamyl alcohol. Pt8Co1/ZSM-5-AT catalyst achieves an extraordinary COL selectivity of 65% at a > 99% CAL conversion efficiency compared with Pt or Co catalysts supported on alkali-treated ZSM-5 zeolite alone. The most remarkable feature of the Pt8Co1/ZSM-5-AT catalyst was that it could be recycled for five times without obvious loss in both CAL conversion and COL selectivity. By combination of the XPS and H_2_-TPR techniques, it was proved that Pt^0^ species and more acid sites were key to concurrently achieve high COL selectivity and CAL conversion efficiency. Due to electron transfer from Co species to Pt atoms, higher electron density of Pt species on the catalyst surface were in favor of the adsorption of C=O bond in CAL and the repulsion of the adsorption of C=C bond. Additionally, more surface acidic sites in the Pt8Co1/ZSM-5-AT catalyst would accelerate COL production and display a high formation of COL in the prolonged hydrogen duration to achieve a high COL selectivity.

## Figures and Tables

**Table 1:** Physical properties of the as-prepared Pt8Co1/ZSM-5-AT, Pt/ZSM-5-AT and Co/ZSM-5-AT catalysts.

Catalyst	Metal Loaded (%) ^a^	Composition (%) ^a^		Specific Area (m^2^·g^−1^) ^b^	Pore Volume (cm^3^·g^−1^) ^c^	Pore Diameter (nm) ^d^	Total Acid Content (µmol·g^−1^) ^e^
		Pt	Co				
Pt/ZSM-5	0.98	100	0	247.4	0.16	0.48	263.9
Pt8Co1/ZSM-5	0.97	88.9	11.1	227.8	0.14	0.47	284.3
Co/ZSM-5	0.97	0	100	242.1	0.17	0.47	173.8

^a^ Metal loaded and Composition results were based on ICP analysis. ^b^ Specific surface area calculated by the BET method. ^c^ Pore volumes were obtained from nitrogen adsorption at P/P_0_ = 0.99. ^d^ Average pore diameter calculated by BJH method. ^e^ Determined by NH_3_-TPD.

**Table 2:** The catalytic results for the liquid-phase selective hydrogenation of CAL over the Pt8Co1/ZSM-5-AT, Pt/ZSM-5-AT and Co/ZSM-5-AT catalysts.

Entry	Catalyst	CAL Conversion (%)	Product Selectivity (%)			
			COL	HCAL	HCOL	Other
1	Pt8Co1/ZSM-5-AT	93.7	68.9	7.7	12.7	10.7
2	Pt/ZSM-5-AT	60.3	53.0	15.6	4.2	27.2
3	Co/ZSM-5-AT	21.9	16.4	44.3	2.8	36.5

Reaction conditions: 200 mg of catalyst, 4 mmol of CAL, P_H2_ = 2 MPa, 20 mL of ethanol, 100 °C, 2 h, stirring rate of 1000 rpm.

## Data Availability

The data that support the findings of this study are available from the corresponding author, upon reasonable request.
